# Editorial: Molecular and epigenetic mechanisms in neuroinflammation and neurodegeneration

**DOI:** 10.3389/fragi.2023.1271714

**Published:** 2023-08-14

**Authors:** Isaac Francos-Quijorna, Elisa Carrasco, Enrique Gabandé-Rodríguez

**Affiliations:** ^1^ Department of Molecular Biology, Faculty of Sciences, Universidad Autónoma de Madrid (UAM), Madrid, Spain; ^2^ Department of Biology, Faculty of Sciences, Universidad Autónoma de Madrid, Madrid, Spain; ^3^ Tissue and Organ Homeostasis Program, Centro de Biología Molecular Severo Ochoa (CBMSO), Consejo Superior de Investigaciones Científicas (CSIC), Madrid, Spain

**Keywords:** aging, neurodegeneration, epigenetics, neuroinflammation, age-associated diseases

## Introduction

The sustained increase in lifespan expectancy achieved during the last years has critically increased the interest in understanding the molecular and cellular mechanisms triggering age-related diseases. Aging is a natural biological process that leads to a progressive decline in physiological functions, making individuals more susceptible to various age-related diseases.

In this Research Topic, Lee et al. review and emphasize the contribution of immunosenescence to the functional decline of the immune system during aging. Among other features, senescent cells typically accumulated in the tissues during aging are characterized by the pro-inflammatory senescence associated secretory phenotype (SASP), contributing to drive local and systemic chronic sterile inflammation through the secretion of multiple cytokines and chemokines. Among the senescent signatures that appear in the major populations of immune cells, previous reports uncovered the decreased extent of type I IFN responses driven by innate immune cells is highlighted as a risk factor for viral infections in aged individuals (Bastard et al., 2020). In parallel, inefficient antibody production by B cells is coupled with age-associated alterations in the function of follicular helper T cells (Almanan et al., 2020), together with defective clearance of senescent cells from the tissues and excessive and promiscuous production of cytotoxic molecules by cytotoxic T cells (Suarez-Álvarez et al., 2017; Pereira et al., 2020). Altogether, these age-associated alterations in different senescent immune cell populations contribute to immune dysfunction during aging. The authors highlight that apart from cell-autonomous responses, the altered aged environment can negatively affect immune cell function. The exposure to viral and bacterial agents exemplified by SARS-CoV-2 can also instigate senescence and contribute to immune cell dysfunction that favours increased multimorbidity, with higher impact on the elderly.

Major age-associated pathologies include neurodegenerative disorders like Alzheimer’s disease (AD). One of the key challenges in diagnosing neurodegenerative diseases is the difficulty in distinguishing between physiological aging and pathological aging. Identifying biomarkers that flag this transition would provide an earlier detection and therapeutic options. In this regard, recent findings point to a prominent role of neuroinflammation in triggering neurodegenerative diseases. While the role of myeloid cells, and specially microglia, was well established, how the adaptive immune system contributes to neurodegenerative diseases is progressively acquiring more relevance in this research field. Indeed, recent findings have evidenced that cytotoxic T cells accumulate in the brain of mouse models and patients with pathologies like AD, Amyotrophic lateral sclerosis or Parkinson’s disease (Carrasco et al., 2022).

In this topic, Cox et al. reviewed the potential of glutamate, a crucial neurotransmitter in the brain, as a biomarker for identifying this pathological transition in AD. During healthy aging, has been reported a decrease in glutamatergic synapses and neurons mainly in the hippocampus, prefrontal cortex and motor sensory areas which in combination with grey matter thinning helps to explain the subsequent cognitive, motor and sensory decline in healthy aging (Segovia et al., 2001; Gasiorowska et al., 2021). However, contrary to healthy aging, research on both human patients and animal models (Minkeviciene et al., 2008; Mura et al., 2012; Hascup et al., 2016; Hascup et al., 2021) showed that early stages of AD are associated with hyperactive glutamate signaling in hippocampus, preceding or coinciding with amyloid and/or tau accumulation, while later stages show reduced glutamate levels due to neuronal loss. Therefore, as Cox et al. highlight in their review, the use of non-invasive imaging methods to monitor glutamate levels, such as cerebrospinal fluid analysis, PET imaging, MRS, and GluCEST might open avenues for early therapeutic interventions aimed at mitigating cognitive decline in AD patients.

Regarding neuroinflammation, Zhuang et al. investigated the involvement of immune cell-related genes and biomarkers in AD development and progression. They used bioinformatic methods to find differentially expressed genes (DEGs) between AD and control samples in three different databases, and by CIBERSORT algorithm they correlated those genes with differentially infiltrated immune cells (DIICs) (Zhuang et al.). They reported that, in line with recent literature data (Heneka et al., 2015; Chen and Holtzman, 2022), several immune cell types, including NK cells, macrophages, dendritic cells, and T cells, showed significant differences in infiltration between AD and control samples. Next, after further narrowed down the focus to immune cell-related DEGs that were highly correlated with specific immune cell types by WGNA analysis, then machine learning methods were employed to identify a set of ten robust immune cell-related genes (CMTM2, DDIT4, LDHB, NDUFA1, NDUFB2, NDUFS5, RPL17, RPL21, RPL26, and NDUFAF2) as potential biomarkers for AD. Then, after validating the expression trends of these genes in peripheral blood samples from AD patients and healthy volunteers by qPCR, authors constructed a diagnostic nomogram by different biomarker combination, as a tool to estimate the probability of disease presence showing promising results in diagnostic accuracy. Finally, they predicted some chemicals, including resveratrol, that could target the identified genes and potentially serve as therapeutic agents in AD treatment using the Comparative Toxicogenomics Database (CTD) and bioinformatic approaches. In conclusion, Zhuang et al.’s report identified several immune-cell-related genes as reliable biomarkers in AD pathogenesis, and their diagnostic monogram offers an important tool for early AD diagnosis despite the need for additional research and validation studies.

Recent research is trying to establish whether neuroinflammation is a process occurring exclusively through the engagement of cell-intrinsic molecular pathways or whether extrinsic or systemic factors contribute to the activation of immune cells. The relevance of this extends beyond basic scientific knowledge as lifestyle habits can influence inflammation and so, can uncover potential targets of pharmacological intervention in these diseases. In this regard, Frausto et al. report in this topic that a mouse model of AD forced to ingest alcohol, develops abnormal intestinal permeability and altered composition of the gut microbiome (intestinal dysbiosis). This correlates with increased systemic levels of the pro-inflammatory cytokine interleukin-6 (IL-6) and increased levels of pro-inflammatory markers in their brains. Moreover, they found a significant correlation of certain bacterial species such as *Lachnospiraceae* NK4A136 and *Clostridium sensu stricto 1* with the altered behaviour and the appearance of disease markers in the brain of these mice (Frausto et al.). These findings align with recent reports that found that altered gut microbiota composition may be an indicator of preclinical AD in patients (Ferreiro et al., 2023).

Finally, in this topic Eric Mayor reviews the potential neurotrophic effects of calorie restriction, exercise practice or intermittent fasting; suggesting that implementation of any or even a combination of these factors could contribute to improve the outcome of patients suffering neurodegenerative diseases. Interestingly, important molecular pathways such as mTOR, NF-ĸB, PGC-1α, MAPK or GSK-3β are targets of these interventions (Mayor). This highlights that research on this field may bring the identification of altered, potentially druggable, molecular pathways in these diseases and not only the characterization of the effects of these interventions that are unfortunately not always easy to implement in these kind of patients.

## Conclusion

This topic includes original papers and review articles summarizing the contribution of different mechanisms ranging from exogenously ingested damaging agents, pro-inflammatory molecules or exacerbated neurotransmitter release to neurodegeneration and age-associated cognitive decline. Moreover, the topic provides with potential therapeutic options that could ameliorate the impaired neuronal performance, which is characteristic of these conditions. On top of that, the readers will also find research on new markers that will eventually help diagnosing these conditions at earlier stages. Future research must deepen into why common mechanisms are altered during the progression of diseases with different aetiology, what could definitely make an impact in patients’ weelbeing.
